# Increasing Regional Anesthesia Use in a Serbian Teaching Hospital through an International Collaboration

**DOI:** 10.3389/fpubh.2017.00134

**Published:** 2017-06-09

**Authors:** Curtis L. Baysinger, Borislava Pujic, Ivan Velickovic, Medge D. Owen, Joanna Serafin, Matthew S. Shotwell, Ferne Braveman

**Affiliations:** ^1^Division of Obstetric Anesthesia, Department of Anesthesiology, Vanderbilt University School of Medicine, Nashville, TN, United States; ^2^Klinika za Ginekologiju I Akuserstvo, Klinickog Centra Vojvodine, Novi Sad, Serbia; ^3^Department of Anesthesiology, SUNY Downstate Medical Center, Brooklyn, NY, United States; ^4^Department of Anesthesiology, Wake Forest School of Medicine, Winston-Salem, NC, United States; ^5^Department of Biostatistics, SUNY Downstate Medical Center, Brooklyn, NY, United States; ^6^Department of Biostatistics, Vanderbilt University School of Medicine, Nashville, TN, United States; ^7^Department of Anesthesiology, Yale University School of Medicine, New Haven, CT, United States

**Keywords:** obstetric anesthesia, neuraxial anesthesia, cesarean delivery, labor analgesia, global health, regional anesthesia

## Abstract

Many low- and middle-income countries (LMICs) report low rates of regional anesthesia (RA) use for cesarean delivery (CD), despite its association with lower maternal major morbidity and mortality. Also, the prevalence of neuraxial analgesia for labor (NAL) is often low in LMICs. We report on the results of a collaboration in clinical education over a multi-year period between Kybele Inc., an international non-profit organization, and Klinicki Centar Vojvodine (CCV), a teaching hospital in Novi Sad, Serbia, to increase RA use for CD and NAL at CCV. From late 2011 through 2015, teams from Kybele participated in annual to biannual didactic conferences and week-long bedside teaching efforts involving obstetric and anesthesia staff from CCV and surrounding hospitals. Ongoing contact occurred at least weekly between Kybele and the host to discuss progress. De-identified quality improvement data on total deliveries, numbers of elective and non-elective CDs, number of vaginal deliveries, type of anesthesia for CD, and the number of NALs were collected. RA use for CD increased to 25% in year 2015 versus 14% in base year 2011 [odds ratio (OR): 2.05; 95% confidence interval (CI): 1.73,2.42; *p* < 0.001]. NAL increased to 10.5% of laboring women in 2015 versus 1.2% in 2011 (OR: 9.6; 95% CI: 7.2, 12.8; *p* < 0.001). Greater increases for RA use during non-elective CD were observed between 2011 and 2015 (1.4 versus 7.5% of total CD; OR: 5.52; 95% CI: 2.63, 8.41; *p* < 0.001) relative to elective CD (12.5 versus 17.5% of total CD; OR: 1.48; 95% CI: 1.23, 1.77; *p* < 0.001). Overall, RA for CD increased during the 4 year collaboration but was not as great as reported in other countries with similar health-care demographics utilizing a similar program. Detailed descriptions of program interventions and barriers to change at CCV are presented.

## Introduction

The use of regional anesthesia (RA) for cesarean delivery (CD) has become widespread in countries in the USA and in Western Europe (1, 2). RA is associated with a lower maternal mortality risk compared to general anesthesia (GA) in both high and low- and middle-income countries (LMICs) (3–7). Despite this recognition, many Eastern European countries report much lower rates of RA use for CD and neuraxial analgesia for labor (NAL) (8, 9). Barriers to implementation in such settings are varied. One report from Georgia cited lack of governmental funding for supplies, lack of familiarity of anesthesia staff with current techniques and evidence-based guidelines, and limited availability of appropriate local anesthetics as possible reasons (10). Another survey of RA use from the Czech Republic cited patient fear over the safety of RA and lack of acceptance by obstetric providers (9), similar to a report of an ongoing collaboration in China between Chinese and American anesthesia providers (11).

## Background and Rationale

Collaborations between non-governmental global health organizations and host institutions in LMICs have been shown to be effective in increasing obstetric RA use (10–13). One report of a single, short-term effort to teach RA techniques in eight hospitals throughout Croatia reported a 50% increase in use of RA for CD and smaller increases in use of NAL (12). Whether these changes were sustained has not been reported. Although single, short-term educational efforts can improve anesthetic performance in some settings, longer collaborative commitments are most often necessary to achieve lasting change (10, 14). Rates of RA use both for CD and labor may regress without ongoing mentoring and support (14).

We report on the rates of RA use at the Klinicki Centar Vojvodine, Klinika za Ginekologiju i Akuserstvo in Novi Sad, Serbia from 2011 to 2015 as part of an ongoing collaboration with Kybele Inc., which began in late 2011. The Klinicki Centar (CCV) is a large tertiary referral obstetric hospital serving the Vojvodine region of Serbia with 6,500 annual births. Kybele, Inc. (www.kybeleworldwide.org) is a 501 (c) (3) non-profit humanitarian organization dedicated to improving childbirth safety through innovative partnerships that provide hands-on bedside and classroom teaching. This study evaluates changes in the rates of RA for CD and NAL before, during, and after expatriate visits and types of anesthesia for both elective and emergency CD over time. We describe our interventions to increase RA use and the barriers to practice change that we encountered.

## Materials and Methods

In September 2011, physicians representing Kybele were invited to participate in a medical conference in Novi Sad, Serbia consisting of 2 days of didactic instruction followed by 1 week of beside instruction in obstetric anesthesia techniques. An ongoing collaboration was then established with one goal to increase the rate of RA use for CD and NAL. The anesthesia staff at CCV consisted of 13 certified specialists and two or three resident trainees who were assigned as part of their residency rotations. Kybele teams consisted of obstetric anesthesiologists, obstetricians, and neonatologists. One anesthesia staff at CCV acted as the primary liaison between Kybele team members, CCV anesthesia and obstetric staff, and the hospital leadership. Annual visits occurred 2012–2014, with biannual visits in 2015, for six visits overall. Visits consisted of a 2-day didactic program of mutually determined topics and 1 week of hands-on clinical instruction for all anesthesia staff. In addition, CCV advertised the program to all certified specialists at hospitals throughout Serbia and the countries of Croatia, Bosnia, and Montenegro.

A curriculum in obstetric anesthesiology was developed over the 2nd and 3rd program years by the Kybele team to provide continuity of instruction material. The material was based on standard, evidence-based obstetric anesthesia practice in well-established training institutions in the USA. This material was available to CCV obstetricians and anesthesia staff both during and between visits. The didactic and clinical instruction programs were each altered from year to year based on anesthesia and obstetric staff input. In addition, informal presentations and discussions occurred frequently between small groups of Kybele team members and CCV obstetric and anesthesia staff during the week long visits. A model consisting of alternating layers of plastic foam of differing densities placed together in a wooden frame was made by members of the Kybele team. This allowed an operator to experience a tactile sensation similar to that encountered clinically when passing an epidural needle into the epidural space. The model was made available primarily to those program participants who had little previous experience in epidural catheter placement.

During each Kybele visit, at least one meeting with the chair of the obstetric department and members of the hospital administration at CCV was conducted to assess the program effectiveness and to suggest new approaches for education and systems change. Suggestions on how to more effectively utilize personnel and clinical resources that would facilitate the performance of RA were made. Upper-level leadership at the hospital expressed support of the ongoing collaboration during these meetings and to Kybele team members during individual encounters. Refinements in processes for best conducting RA were suggested.

During each visit, local media outlets interviewed both Kybele and CCV leaders, and the benefits of RA use in obstetrics were presented. The interviews, which were broadcast throughout Vojvodine, Serbia, stressed the collaboration between the host and Kybele teams. A patient information brochure, which explained anesthetic choices for CD, was made available to all patients upon admission to the hospital the night before elective cesarean section.

During the periods between visits, telephone conversations occurred weekly between one Kybele team leader and the principal liaison to discuss questions regarding patient management, administrative issues (most often, how best to increase RA use), and obstetrician questions on providing clinical anesthesia care. These discussions provided the basis for altering the approaches used by the Kybele team from visit to visit.

The Institutional Ethics Committee of Klinicki Centar Vojvodine gave permission for the collection of patient de-identified data for a quality improvement assessment of the ongoing collaborative effort. To examine the shorter-term impact following each Kybele visit, data on the use of RA for CD were collected 1 week before a visit (R1), the week during (R2), and at one (R3) and two (R4) weeks, and two months (R5) following a Kybele visit.

Multiple logistic regression was used to estimate the relative changes in the odds of obstetric RA use in the week before, during, and after visitation by Kybele, adjusting for a visit. Thus, the fraction of CDs (during those weeks) in which RA was used was the dependent variable and week sequence and visit were treated as categorical independent variables.

A second pair of simple logistic regression analyzes was used to assess the odds ratios (ORs) of RA for CD and NAL with calendar year (2011–2015) as the categorical independent variable. This analysis included all CDs by calendar year, whereas the analysis above considered only those in the weeks before, during, and after a visit by study investigators. Long-term-sustained effects on RA use for CD for each year were assessed in the period 2012–2015 relative to year 2011 as the base year.

Finally, the association between elective versus non-elective CD and the rate of RA use was evaluated using a multiple logistic regression analysis adjusting for categorical calendar year and elective versus non-elective CD and their interaction. The interaction was used to assess whether the OR for RA use in 2015 versus 2011 varied by elective versus non-elective status. All ORs were summarized using Wald-type 95% confidence intervals (CIs) and *p*-values.

## Results

The mean annual number of deliveries was 6,382 (range 6,084–6,570) and mean yearly number of CDs was 1,964 (range 1,794–2,043) during the study period. The use of RA for CD increased significantly over time compared to 2011 (Table 1). In 2012 and 2013, the relative odds of RA use were greater by 18% (95% CI: −1, 41; *p* = 0.067) and 39% (95% CI: 16, 65; *p* < 0.001), respectively. In 2014 and 2015, the relative odds were greater by at least twofold (2014 OR: 2.20; 95% CI: 1.87, 2.60; *p* < 0.001. 2015 OR: 2.05; 95% CI: 1.73, 2.42; *p* < 0.001). For elective cases, RA for CD increased from 12.5% of total CD in 2011 to 17.5% in 2015 (OR: 1.48; 95% CI: 1.23, 1.77; *p* < 0.001). For non-elective cases, RA for CD increased from 1.4 to 7.5% of total CD from 2011 through 2015 (OR: 1.48; 95% CI: 1.23, 1.77; *p* < 0.001). Use of RA for non-elective cases increased more than for elective cases (interaction OR: 3.74; 95% CI: 2.36, 5.92; *p* < 0.001).

**Table 1:** Yearly percentage of cesarean deliveries (CDs) performed under regional anesthesia (RA) and percentage of vaginal deliveries (VD) with neuraxial analgesia for labor (NAL) at Clinical Center Vojvodine.

Year	Percentage of CD under RA	Percentage of elective CD under RA	Percentage of non-elective CD under RA	Number of CDs	Percentage of VD under NAL	Number of VDs
2011	14.0	12.5	1.4	1,794	1.2	4,295
2012	16.1	13.7	2.4	2,043	3.2	4,312
2013	18.4	15.1	3.3	1,998	5.8	4,330
2014	26.4	19.0	7.4	2,004	8.0	4,500
2015	25.0	17.5	7.5	1,996	10.5	4,574

The use of NAL increased significantly over time compared to base year 2011 (Table 1). In 2012 and 2013, the relative odds of NAL use were greater by 2.7-fold (95% CI: 1.96, 3.72; *p* < 0.001) and 5.0-fold (95% CI: 3.7, 6.8; *p* < 0.001), respectively. In 2014 and 2015, the relative odds were greater by 7.1-fold (95% CI: 5.3, 9.5; *p* < 0.001) and 9.6-fold (95% CI: 7.2, 12.8; *p* < 0.001), respectively.

### Effects of Kybele Visits 2012–2015

The immediate impact of a visit on the use of RA for CD showed a large positive effect that decreased over time. After adjusting for the visit sequence (one visit in 2012–2014 and two visits in 2015), the relative odds of RA use during the week of a Kybele visit were 2.57 times greater (OR: 2.57; 95% CI: 1.60, 4.14; *p* < 0.001) than the base week preceding a visit. Although use regressed over time, RA use was consistently higher than for the base comparison week: the OR was 1.96 (95% CI: 1.22, 3.16; *p* = 0.006) during the week after the visit, but by week two and eight, the ORs were 1.66 (95% CI: 1.01, 2.72; *p* = 0.046) and 1.70 (95% CI: 1.04, 2.79; *p* = 0.034), respectively. After adjusting for week, year was not significantly associated with the relative odds of RA use during those weeks (except for June, 2015 versus 2012) (Tables 2 and 3).

**Table 2:** Number of cesarean deliveries (CDs) performed under regional anesthesia at Clinical Center Vojvodine around Kybele visits and overall, Calendar Years 2012–2015.

	Visit year				
Interval	2012	2013	2014	6/2015*	9/2015
R1 (%)	13	21	22	17	21
# C/D	38	33	45	35	33
R2 (%)	31	42	38	40	38
# C/D	29	36	37	42	39
R3 (%)	24	39	17	43	33
# C/D	33	38	36	44	45
R4 (%)	24	18	30	39	33
# C/D	34	38	33	36	36
R5 (%)	28	20	25	39	34
# C/D	32	41	36	31	38

*p = 0.016 compared to the corresponding time intervals in 2012.

*R1 = week before a Kybele visit*.

*R2 = week during a Kybele visit*.

*R3 = week after a Kybele visit*.

*R4 = two weeks after a Kybele visit*.

*R5 = two months after a Kybele visit*.

**Table 3:** OR for regional anesthesia for cesarean delivery (CD) adjusting for week around a Kybele visit.

	OR	95% CI	*p*-value
Visit: 2013	1.19	0.73, 1.94	0.475
Visit: 2014	1.15	0.71, 1.88	0.567
Visit: June, 2015	1.78	1.11, 2.85	0.016
Visit: Sept., 2015	1.51	0.94, 2.42	0.090
Week: R2	2.57	1.60, 4.14	<0.001
Week: R3	1.96	1.22, 3.16	0.006
Week: R4	1.66	1.01, 2.72	0.046
Week: R5	1.70	1.04, 2.79	0.034

*The reference week is the week before a Kybele visit (R1), and the reference year/month is 2012*.

*OR, odds ratio; 95% CI, 95% confidence interval*.

*R2 = week during a Kybele visit*.

*R3 = week after a Kybele visit*.

*R4 = two weeks after a Kybele visit*.

*R5 = two months after a Kybele visit*.

## Discussion

During the CCV-Kybele collaboration, the use of NAL increased from 1.2 to 10% and the use of RA for CD increased from 14% to approximately 25%, which plateaued over the last 2 years of the study period. Reports from both high and LMIC countries show that RA use is associated with significant reductions in anesthetic-related maternal morbidity and mortality compared to GA (3, 4, 6, 7). Accordingly, the 4 year on going collaboration between Kybele and CCV focused on increasing RA use and our results showed progress in achieving that goal, with significant increases in the use of RA for CD and NAL. These changes have been sustained over time despite decreases in use of RA that occurred in the immediate 2-month period following training visits. Kopic et al. reported increases in RA for CD and NAL following their program in Croatia, a neighboring Eastern European country similar in health-care demographics to Serbia (12). The use or RA for CD at the study institutions increased from 20% before their one time, country-wide, multi-institutional intervention to 34% following the program, a rate similar to that reported in year 2015 of the collaboration at CCV. Ninidze et al. (10) described better success in their collaboration of yearly program visits over 4 years to five participating hospitals in Georgia, another country with health-care demographics similar to Serbia. The importance of repeated visits over time was emphasized in this study, as the three hospitals who were visited four times reported increases in RA use for CD from approximately 20 to 80%, while the two hospitals visited only twice reported more modest increases from approximately 25 to 50%.

The present study sought to further define short-term variations in practice that occur temporally around a training intervention. During all visits, the use of RA for CD increased substantially compared to the baseline immediately before a visit, with less use of RA over time in the several weeks following an intervention. A diminishing effect of the shorter- and longer-term impact of interventions occurred; the results of the two visits in 2015 were similar to those in 2014. This outcome is not unexpected. Kybele team members provided additional patient care over that provided by CCV staff, and staff were enthusiastic in working with Kybele team members during the week visit. However, by 2015, all of anesthesiologists had participated in at least one previous program, which might have diminished the effects of additional 1 week educational efforts. Further improvement would most likely require Kybele visits of greater than 1 week and more individually directed education efforts.

The unexpected finding of a larger percentage increase in the use of RA in non-elective CDs compared to a smaller overall increase in elective cases suggests that patient preference may be a significant factor in determining anesthetic choice. In cases of elective, repeat CD, patients will have most often experienced successful GA with a prior CD and will be familiar with it, versus lack of experience with RA. Stourac et al. (9), Kopic et al. (12), Olufolabi et al. (13), and Schnittger (15) all cited patient fear of being awake during a RA as a major factor in patient reluctance to choose RA. In year three of our effort (2013), a patient information card was made available to all patients who underwent elective CD, as they were admitted the night before an elective CD. Reference to it was made during the preoperative interview by those anesthesia personnel, who discussed anesthetic options immediately before CD. While this may have been an important factor in the increase in RA for CD between 2013 and 2014, its effectiveness may have been reduced if used by anesthesia personnel less than enthusiastic about performing RA.

While patient education regarding anesthesia for repeat CD in the immediate preoperative period is important in increasing RA use, such efforts should be considered during routine predelivery obstetric visits. Obstetricians often have a major influence over patient choice of anesthesia (10). Obstetrician preference may have a larger effect than that of the anesthesiologist in the choice of RA versus GA and efforts directed at obstetrician education to adequately answer patient questions concerning RA may be as important as those directed toward patients in increasing patient acceptance (10). An added benefit is that it may also overcome obstetrician reluctance to RA use (9, 11–13, 15). Ninidze et al. (10), Kopic et al. (12) Olufolabi et al. (13), and Schnittger (15) all cited obstetrician reluctance to operating on an awake patient as a potential barrier to use of RA for CD. We did not survey the obstetricians at CCV as to reasons why they might object to RA for CD. Our educational efforts were during conferences at which attendance was voluntary; many obstetricians did not participate.

We found that NAL use increased substantially during our multi-year program but is significantly less than the 60% rate cited for developed countries such as the USA (1). Our multi-year collaboration achieved greater success than that reported by Kopic et al. (12) following their one-time intervention (from 1.2 to 2.3%), but the overall rate of approximately 10% we saw in 2015 is considerably less than the 45% rate reported by Ninidze et al. (10) at the end of their multiyear intervention. However, the initial rate of NAL was considerably higher (20%) in the hospitals where Ninidze et al. worked than that at CCV, suggesting that the infrastructure for NAL (not described in their report) was already established before the training intervention.

Howell recently opined that the success of multi-national collaborative efforts is dependent on identifying a local partner in the host institution committed to change, presentation of new concepts in a way acceptable to recipients, team members who are committed to engage with local practitioners, and continuous follow-up between educational visits (14). Reed et al. noted that technical training, carefully implemented in cooperation with the host, with repeated efforts during and in between site visits to overcome obstacles over a significant period of time are necessary for successful change (16). Our program incorporated most of these approaches. The program at CCV collaborated with a local host enthusiastic in achieving the mutually determined goals. Kybele, the host, and the senior leadership at CCV committed to a multi-year program. A curriculum that responded to the educational needs of the staff at CCV was developed. Kybele members worked closely with staff at CCV in uniformly positive ways and most expressed enthusiasm for the interventions. Kybele team members and the local liaison communicated regularly during the intervals between visits. The methods described in the multiyear effort reported by Ninidze et al. (10) employed these principles but with greater success.

The lack of significant increases in RA for CD from 2014 to 2015 and only modest absolute increases in NAL over the 4 year period are probably due to several structural and process factors unique to the practice setting at CCV. The principal liaison between Kybele and CCV was not the chief of the department of anesthesia. Although the chief of anesthesia at CCV supported the collaboration, the Kybele team did not meet with her regularly during visits or communicate with her between visits. Kybele’s ability to approach anesthesia providers who did not increase RA use was thus limited. Also, we were unable to secure her permission to display de-identified charts of each anesthesiologist’s RA use over time. This technique has been shown to be effective by others in implementing provider behavioral change in other health-care settings (17). In addition, anesthesia personnel felt obligated to continuously monitor NAL delivered by continuous infusion, thus limiting the availability of personnel to provide NAL, especially during call hours. We were unable to change this practice despite presenting that NAL by continuous infusion without continuous monitoring by anesthesia personnel is routine in the USA.

Early in the collaboration the Kybele team noted that all anesthesia department members were available for work during the day 8-h workday, with only a single staff person for call coverage beginning midafternoon. Thus, many persons were available for work during the regular 8-h workday, but very few after that time. This staffing model limits personnel available to perform NAL, especially since the call person performs all anesthesia for CD as well. We were unable to change this staffing model.

Finally, although the head of the hospital at CCV was enthusiastic about our collaborative effort, his enthusiasm may not have been shared by others. Some obstetricians were enthusiastic in interacting with Kybele members and welcoming of changes in anesthesia care for their patients, but others were not. We were unable to obtain the leadership’s support for changing the anesthesia staffing model as noted above. We were also unable to obtain support for placing patient information brochures on choices of anesthesia for vaginal and CD in obstetricians’ offices.

## Conclusion

We report modest success of the Kybele CCV collaboration in increasing the use of RA for CD and for pain relief during labor. The increase in RA use was not as great as reported by others in other countries with similar health-care demographics but increases in the use of NAL are similar. Despite nearly weekly interaction between Kybele team members following 1-week interventions one and two times per year and a largely enthusiastic host, rates of RA use for CD at CCV are only 1/3 of those in Western European and North American practices (7, 9, 12).

Although barriers to change at CCV are similar to that reported by others (acceptance by anesthesia personnel, acceptance by obstetrical personnel, enthusiasm by hospital leadership that translate into staff enthusiasm, and improved patient education prior to delivery and in the immediate delivery period), factors unique to CCV (anesthesia staffing model, ability to target individual obstetrician and anesthesiologists for education efforts, better ways of providing education to patients) will need to be changed before further progress can be made. It is likely that Kybele team visits lasting greater than 1 week will be required.

## Ethics Statement

Institutional Ethics Committee of Klinickog Centra Vojvodine gave permission for collection of patient de-identified data for a quality improvement assessment of the ongoing collaborative effort.

## Author Contributions

CB, BP, IV, MO, JS, MS, and FB, all made substantial contributions to the conceptual design of the work, in acquiring the data, and in its analysis. All helped draft the work and revised it critically. All approve this copy for publication. All agree to be accountable for all aspects of the work in ensuring its accuracy and integrity.

## Conflict of Interest Statement

The authors declare that the research was conducted in the absence of any commercial or financial relationships that could be construed as a potential conflict of interest. The reviewer, NG, and the handling editor declared their shared affiliation, and the handling editor states that the process nevertheless met the standards of a fair and objective review.
